# 

**Published:** 1915-03

**Authors:** 


ESTABLISHED JULY, 1885

PUBLISHED MONTHLY AT AUSTIN, TEXAS, BY

MRS. F. E. DANIEL, - - - Publisher and Managing Editor

**Subscription, $1.00 a Year, in Advance. - - - - Single Copies, 10 Cents**

Entered at the Postoffice at Austin, Texas, as second class mail matter

